# Epigenetics as an answer to Darwin’s “special difficulty”

**DOI:** 10.3389/fgene.2014.00321

**Published:** 2014-09-12

**Authors:** Brian R. Herb

**Affiliations:** Center for Epigenetics and Department of Medicine, Johns Hopkins University School of MedicineBaltimore, MD, USA

**Keywords:** epigenetics, evolution, genomics, developmental plasticity, eusociality, behavior, pheromones

## Abstract

Epigenetic modifications produce distinct phenotypes from the same genome through genome-wide transcriptional control. Recently, DNA methylation in honeybees and histone modifications in ants were found to assist the formation of caste phenotypes during development and adulthood. This insight allows us to revisit one of Darwin’s greatest challenges to his natural selection theory; the derivation of multiple forms of sterile workers within eusocial species. Differential feeding of larvae creates two distinct developmental paths between queens and workers, with workers further refined by pheromone cues. Flexible epigenetic control provides a mechanism to interpret the milieu of social cues that create distinct worker sub-caste phenotypes. Recent findings suggest a distinct use for DNA methylation before and after adult emergence. Further, a comparison of genes that are differentially methylated and transcriptionally altered upon pheromone signaling suggests that epigenetics can play a key role in mediating pheromone signals to derive sub-caste phenotypes. Epigenetic modifications may provide a molecular mechanism to Darwin’s ”special difficulty” and explain the emergence of multiple sub-phenotypes among sterile individuals.

A major defining feature of eusocial species is the division of labor among phenotypically distinct castes (Winston, 1987). The evolution of such a system required the eventual partitioning of all reproductive tasks to a single individual, leaving the remaining tasks to sterile relatives. This arrangement, however, posed a great challenge to Darwin’s theory of natural selection (Darwin, 1859). The bedrock of his theory was that successful individuals passed on traits to the next generation. How then, could a sterile individual possess traits distinct from reproductive individuals and not have the means to pass them on? Darwin wrote:

… one special difficulty, which at first appeared to me insuperable, and actually fatal to the whole theory. I allude to the neuters or sterile females in insect-communities; for these neuters often differ widely in instinct and in structure from both the males and fertile females, and yet, from being sterile, they cannot propagate their kind.

Lacking a through understanding of the underlying genetics, Darwin nonetheless had the great insight that these sterile workers, being related to the reproductive member of the colony, can ensure the survival of their species by helping the colony as a whole. This insight did not fully answer the challenge, because he goes on to marvel, not at the existence of sterile workers *per se,* which he equates to the trait divergence between males and females, but rather how can multiple sub-phenotypes of sterile workers arise:

The great difficulty lies in the working ants differing widely from both the males and the fertile females in structure, as in the shape of the thorax, and in being destitute of wings and sometimes of eyes, and in instinct. As far as instinct alone is concerned, the wonderful difference in this respect between the workers and the perfect females would have been better exemplified by the hive-bee.

Darwin’s curiosity might have been further heightened if he knew that for most social insects the reproductive and sterile females are genetically identical. Phenotypic difference in the absence of genetic difference falls in the realm of epigenetics, which is the study of heritable information other than the DNA sequence itself. Epigenetic information can be stored in the molecular form as methylation on the cytosine base of DNA or a variety of modifications to histone tails (Kouzarides, 2007; Jones, 2012). These epigenetic modifications play a key role in tissue development where drastically different organs are derived from the same genome (Irizarry et al., 2009). Here we explore the role of epigenetic modifications in caste determination and propose that epigenetic machinery is important to derive the multiple forms of sterile workers that vexed Darwin so long ago.

Honeybees (*Apis mellifera*) have unique control over the developmental fate of the females in the colony through differential feeding of the larvae and pupae. A nutrient rich diet of royal jelly produces a reproductive queen, and the absence of such diet produces facultatively sterile workers. This royal jelly contains royalactin, a potent activator of p70 s6 kinase that increases ovary development and shortens development time (Kamakura, 2011). Queen development is marked by an increase in Tor activity during the third to fifth instars, stimulating growth and increased metabolism. Increased Tor activity occurs at the developmental time point when queens and workers diverge into two irreversible paths, permanently locking in caste differences. RNAi knockdown of Tor causes larvae to prolong development, reduce growth and ultimately emerge as workers, even on a diet of royal jelly (Patel et al., 2007; Mutti et al., 2011).

While honeybee hives have a single queen that lays millions of eggs over her 2–3 year lifespan, thousands of workers perform the remaining tasks. The typical adult worker will first act as a nurse to raise the young, attend to the queen, and clean combs for the next generation. About 8 days post-emergence, the worker will transition into foraging tasks, which are metabolically taxing and accelerate physiological decline (Winston, 1987). However, the division of labor is dependent on the needs of the colony, and individuals within the hive are able to communicate these needs either through direct contact or by pheromones. Queen mandibular pheromone (QMP) is emitted by the queen in order to recruit nurse bees to care for her, suppress ovary growth in workers and discourage workers from raising a new queen. The brood translates its own needs by emitting brood pheromone (BP) to stimulate nurse bees to feed and care for the brood. BP also influences nurse bees to delay the transition into foraging, and existing foragers to skew their collecting toward the protein source pollen (Slessor et al., 2005). Workers further refine their tasks by physically interacting with fellow workers and recruiting them to specific tasks based on the needs of the hive (Seeley et al., 1998). Foragers themselves can also suppress nurse bees from foraging by emitting Ethyl oleate (Leoncini et al., 2004). So it is in this environment of constant signals that the worker bee refines her role throughout life. These signals form a basis for unlocking multiple phenotypes, the marvel of Darwin 150 years ago.

Honeybees use diet and social cues to separate genetically similar females into distinct roles, but what is the underlying molecular mechanism that integrates environmental stimuli and solidifies phenotype? Lacking a strong genetic candidate, epigenetic modifications can drive differentiation of multiple phenotypes as seen with cellular lineages in blood (Ji et al., 2010). The beauty of epigenetic mechanisms is that they can assist in maintaining a particular transcriptional state by storing information in the form of temporary chemical tags at the level of DNA itself. DNA methylation and histone modifications have been thoroughly studied in mammals and are known to play a major role in development and disease (Ho and Crabtree, 2010; Hansen et al., 2011). Genome-wide epigenetic modifications, like DNA methylation, can be context specific depending on their placement relative to genes and enhancers. A unique combination of DNA methylation and histone modifications in the promoter of a given gene can have a persistent repressive effect when these marks are bound by proteins that in turn establish larger protein complexes that as a whole suppress transcription. A good example of this process occurs during mammalian differentiation where pluripotency genes such as *OCT4* and *NANOG* are silenced by methylation of H3K9 by G9a, which in turn leads to condensing of chromatin by HP1 binding and eventual DNA methylation (Feldman et al., 2006; Smith and Meissner, 2013). This step-wise change in epigenetic modifications indicates different degrees of repression that become increasing resistant to activation. These epigenetic modifications can be reversed, but require persistent signals, such as the expression of the reprograming factors *OCT4*, *SOX2*, *MYC,* and *KLF4* to derive induced pluripotent stem cells (iPS; Doi et al., 2009). Another example of dynamic epigenetic change is during the activation of the *pS2* gene upon estrogen signaling. Time-course experiments showed active demethylation of DNA and recruitment of chromatin remodeling proteins to the site of the *pS2* gene after estrogen signaling (Metivier et al., 2008). In addition to chemical modifications to DNA and histone tails, RNA itself can provide temporal control of gene expression through the binding of non-coding RNAs to DNA, proteins or other RNAs. Non-coding RNAs can organize chromatin structure on a large scale as evidenced by X chromosome inactivation by the ncRNA *Xist*, or control local expression in the case of the ncRNA *Air* interacting with the histone methyltransferase G9a to silence the *Slc22a3* gene during development (Nagano et al., 2008; Mercer and Mattick, 2013). Studies investigating DNA methylation differences between worker subcastes in honeybees (Herb et al., 2012) and between queens and workers in ants (Bonasio et al., 2012) have found that many differentially methylated genes are involved in non-coding RNA processing, suggesting a role for non-coding RNA in caste determination. While the fundamental role and scope of non-coding RNA in mammalian development is established (Mattick, 2011), the impact of non-coding RNA in social insects is just starting to be understood (Bonasio, 2012; Humann et al., 2013), therefore the focus of this perspective will only include epigenetic modifications that have been mapped genome-wide, namely DNA methylation and histone modifications. Overall, the temporal control of epigenetic modifications enforces a specific transcriptional state by storing information at the level of the DNA itself, which remembers that state until a new stimuli is encountered.

Only recently has the importance of epigenetics in social insects been appreciated through the discovery of DNA methylation in many species (Bonasio et al., 2010; Beeler et al., 2014). Social insects are an ideal test ground for studying the role of epigenetic mechanisms because they can derive multiple behavioral phenotypes from the same genome. The first major clue that epigenetics played a role in queen/worker differentiation came soon after the complete sequencing of the honeybee genome in 2006 (Consortium, 2006) when the presence of DNA methyltransferase enzymes confirmed a functional DNA methylation system (Wang et al., 2006). Kucharski et al. (2008) knocked down Dnmt3 in larvae and found that regardless of diet, most knockdowns developed queen features. This initial result inspired a genome-wide search for functional DNA methylation differences between queens and workers that resulted in three major studies that interrogated three developmental time points; larvae (Foret et al., 2012), adult emergence (Herb et al., 2012), and advanced age adults (Lyko et al., 2010). While differences between queens and workers were found across the genome in larvae and advanced age adults, there were no statistically significant differences at the time of adult emergence (**Figure 1A**). While these studies take different approaches to find regional changes in DNA methylation, the large number of differences found in larvae compared to the complete absence of differences between queens and workers strongly suggest that DNA methylation is required to maintain queen/worker differences during the larval stage, but are not required to separate newly emerged queens and workers when morphological differences are irreversible. DNA methylation appears to target many genes of the Tor pathway (Mutti et al., 2011; Foret et al., 2012), which has been implicated in queen worker developmental differentiation (Patel et al., 2007). It is possible that DNA methylation assists in maintaining the activation of Tor pathway genes caused by royal jelly. Returning to Darwin’s difficulty, we see that DNA methylation can assist in maintaining separate transcriptional programs for queens and workers in honeybees, providing a mechanism for maintaining caste differences. Further proof that epigenetic mechanisms help produce alternative phenotypes is illustrated by the finding that histone modifications, in particular H3K27ac, differentiate major from minor workers in the carpenter ant *Camponotus floridanus* (Simola et al., 2013). The remarkable size difference between ant worker sub-castes was particularly striking to Darwin and this result illustrates that epigenetic modifications, including histone modifications, can help produce multiple sterile worker phenotypes (Darwin, 1859). This example from ants bolsters the idea of epigenetic modifications solidifying differences initiated by diet during development, but how do epigenetic modifications help individuals navigate transitions throughout adult life as seen above with nurses and foragers?

**FIGURE 1:**
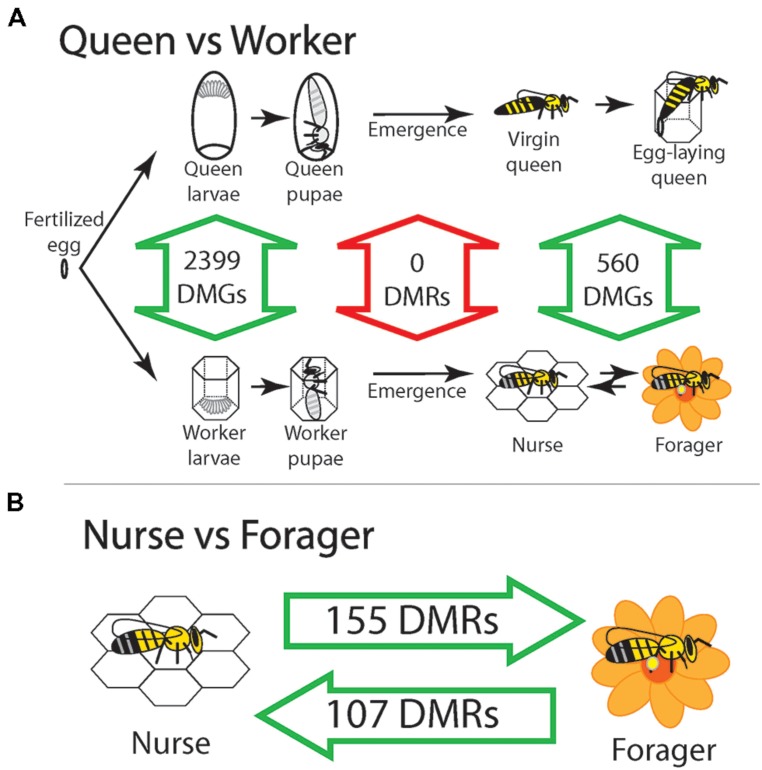
**Honeybee castes are distinguished by methylation except at the time of emergence. (A)** Summary of multiple studies show that DNA methylation separates queens and workers during larval development, but this difference disappears by the time they emerge as adults. Number of differentially methylated genes (DMGs) and differentially methylated regions (DMRs) are from the following studies – larvae: (Foret et al., 2012), emergent queens and workers and adult nurses and foragers: (Herb et al., 2012) and adult queens and workers: (Lyko et al., 2010). **(B)** Reversible differences between nurses and foragers show that DNA methylation is used to temporarily lock in sub-caste phenotypes within the life time of workers as they transition between tasks. Intra-caste changes in DNA methylation between nurses and foragers during adulthood could explain inter-caste differences observed between adult queens and workers. Since queens and workers are the same at emergence, it is possible that DNA methylation is no longer needed to maintain the irreversible morphological difference between queens and workers, and any difference in DNA methylation observed between adult queens and workers are simply a result of intra-caste changes in adults.

While worker bees generally transition from nursing to foraging tasks over their lifetime, the timing of this transition and exact task they perform at any given point along this continuum is refined by social cues within the hive (Slessor et al., 2005). Specific pheromones can elicit a change in expression of hundreds of genes and recruit workers to a task or delay their transition into a new task (Grozinger et al., 2003; Alaux et al., 2009). Although powerful, these signals must be regarded in the context of the hive, where the organization of the brood in the center and the storage of pollen and honey on the periphery create “task zones.” Workers are born into a region where the queen is actively laying eggs and pheromones from the queen and brood are strongest and influence the newborn worker to assume nursing tasks. As workers age, they encounter returning foragers that present recruitment signals to elicit the nurses to transition into new roles (Winston, 1987; Whitfield, 2003). However, if upon the first interaction with a returning forager, a nurse flew out of the nest and began collecting nectar, or inversely upon a whiff of queen pheromone foragers began caring for the brood, the hive would be in chaos. Instead, tasks are performed for continuous periods and a transition requires repeated cues to initiate. The flexible control that epigenetics offers is an ideal mechanism for interpreting social cues within the hive and provide temporal control over gene expression.

Workers generally start their adult lives performing nursing tasks and transition into foraging tasks, but it is possible to revert foragers back to nursing tasks if the need arises (Amdam et al., 2005). This reversion schema includes two types of nurses, one set that has always performed nursing tasks, and one set that has had foraging experience that reverts back to nursing. When age-matched nurses, foragers and reverted nurses were compared, hundreds of differentially methylated regions (DMRs) distinguished these phenotypes. Incredibly 57 DMRs followed the behavioral reversion where DNA methylation levels changed during the nurse to forager transition, and changed back to original nurse levels during the reversion (**Figure 1B**). Genes associated with these 57 reversion DMRs had far reaching developmental and gene regulatory functions, including multiple genes containing DEAD-box helicase domains that act through chromatin remodeling to affect global gene expression. Importantly, distinct nurse and forager specific epigenetic signatures were identified, demonstrating that specific levels of DNA methylation in the brain are required to form sub-caste phenotypes. This study was also the first evidence of reversible methylation underlying a behavioral trait and demonstrates the flexible control epigenetic modifications can bestow on phenotype (Herb et al., 2012).

If pheromones and other social interactions impact the epigenome, then the socially defined task is remembered at the level of the genome itself. This also ensures an intrinsic “buffering” against instantly changing task upon the random encounter with workers performing different tasks. This buffering would ensure that only persistent social cues, reflective of the true needs of the hive, would cause the worker to switch tasks. This is not meant to detract from or oversimplify the complex network of social cues in the hive that organize labor in such an efficient way. Rather, coupling the flexible control of epigenetics with the spatial and social cues in the hive establishes a framework for understanding how workers can achieve stabilized sub-caste phenotypes. If true, then genes under the influence of pheromones should also be regulated by epigenetic modifications. The influences of two major pheromones, QMP and BP, have been studied at the genome level using gene expression microarrays. Key genes *POE*, active in neuro-development, and the chromatin remodeling genes *HCF* and *ISWI* are all chronically regulated by QMP (Grozinger et al., 2003) and are also differentially methylated between nurses and foragers (Herb et al., 2012). In addition, genes regulated by BP and those differentially methylated between nurses and foragers (Alaux et al., 2009) share functional enrichment of helicase and nucleoside binding genes. BP influences the expression of the developmental genes *WIT*, *UNR*, and *BICD* (Alaux et al., 2009) and these genes are associated with reversible methylation between nurses and foragers (Herb et al., 2012). Epigenetic control of a core group of master regulatory genes may be sufficient to help maintain pheromone-induced phenotypes and help stabilize the division of labor in the hive. For example, the chromatin-remodeling gene *ISWI*, which is regulated by QMP and differentially methylated between nurses and foragers, remodels nucleosomes around gene promoters (Sala et al., 2011). The action of Iswi may facilitate the large-scale gene expression differences observed during the nurse to forager transition (Whitfield, 2003).

Social insects are masters at controlling the division of labor within their colonies to maximize efficiency and react to changing environmental conditions. However, the evolution of the sterile worker proved troublesome to Darwin, who struggled to incorporate the existence of multiple phenotypically distinct sterile workers in his natural selection theory that emphasized passing traits directly to the next generation. Through differential feeding and social cues, the colony as a whole has evolved numerous mechanisms to fine-tune the phenotypes of sterile workers to obtain an efficient division of labor. We can now integrate the action of epigenetic machinery to the evolution of social insects. Epigenetic information stabilizes phenotype and provides a mechanism for deriving multiple castes from the same genome. This extra layer of information works with established signaling pathways and regulatory programs to lock in gene expression patterns and interpret external stimuli. DNA methylation appears to play two major roles, distinguishing queens and workers during development and defining sub-castes within the lifetime of worker bees. Further, based on the limited presence of methylation across the honeybee genome, it seems that methylation has been reserved to act on select genes that have far reaching effects. These key genes are regulated by epigenetics but are initiated by social cues within the hive, directing the division of labor. As seen with histone modifications in ants and DNA methylation in honeybees, epigenetics plays an important role in social insects. Perhaps it will bear out that utilizing epigenetic machinery to derive additional worker phenotypes was critical to the evolution of eusociality in insects.

## Conflict of Interest Statement

The author declares that the research was conducted in the absence of any commercial or financial relationships that could be construed as a potential conflict of interest.
